# Geographic Information System-based Screening for TB, HIV, and Syphilis (GIS-THIS): A Cross-Sectional Study

**DOI:** 10.1371/journal.pone.0046029

**Published:** 2012-10-02

**Authors:** Neela D. Goswami, Emily J. Hecker, Carter Vickery, Marshall A. Ahearn, Gary M. Cox, David P. Holland, Susanna Naggie, Carla Piedrahita, Ann Mosher, Yvonne Torres, Brianna L. Norton, Sujit Suchindran, Paul H. Park, Debbie Turner, Jason E. Stout

**Affiliations:** 1 Department of Medicine, Duke University Medical Center, Durham, North Carolina, United States of America; 2 Wake County Human Services, Raleigh, North Carolina, United States of America; 3 Wake County Community Services, Raleigh, North Carolina, United States of America; McGill University, Canada

## Abstract

**Objective:**

To determine the feasibility and case detection rate of a geographic information systems (GIS)-based integrated community screening strategy for tuberculosis, syphilis, and human immunodeficiency virus (HIV).

**Design:**

Prospective cross-sectional study of all participants presenting to geographic hot spot screenings in Wake County, North Carolina.

**Methods:**

The residences of tuberculosis, HIV, and syphilis cases incident between 1/1/05–12/31/07 were mapped. Areas with high densities of all 3 diseases were designated “hot spots.” Combined screening for tuberculosis, HIV, and syphilis were conducted at the hot spots; participants with positive tests were referred to the health department.

**Results and Conclusions:**

Participants (N = 247) reported high-risk characteristics: 67% previously incarcerated, 40% had lived in a homeless shelter, and 29% had a history of crack cocaine use. However, 34% reported never having been tested for HIV, and 41% did not recall prior tuberculin skin testing. Screening identified 3% (8/240) of participants with HIV infection, 1% (3/239) with untreated syphilis, and 15% (36/234) with latent tuberculosis infection. Of the eight persons with HIV, one was newly diagnosed and co-infected with latent tuberculosis; he was treated for latent TB and linked to an HIV provider. Two other HIV-positive persons had fallen out of care, and as a result of the study were linked back into HIV clinics. Of 27 persons with latent tuberculosis offered therapy, nine initiated and three completed treatment. GIS-based screening can effectively penetrate populations with high disease burden and poor healthcare access. Linkage to care remains challenging and will require creative interventions to impact morbidity.

## Introduction

In the United States, microepidemics of human immunodeficiency virus (HIV) and tuberculosis (TB) persist with high prevalence in specific areas, and rates of syphilis are on the rise [1], [2], [3]. Propagation of these infections has been in part attributed to high-risk populations living in neighborhoods with poor health care access and utilization [4], [5], [6]. Broad screening measures such as Centers for Diseases Control (CDC)recommendations to routinely screen persons aged 13–64 for HIV may be less effective than more targeted strategies [7]. Engaging difficult-to-access, underserved populations will require innovative integrated strategies in order to move the U.S. closer towards eradication of HIV, TB, and syphilis.

One such targeted strategy has been the integration of surveillance information and geographic data to identify “hot spots” for disease transmission. Geographic information systems (GIS) analysis has been used to better understand transmission dynamics of syphilis, HIV and tuberculosis [8], [9], [10], [11], [12]. Rationale for a GIS-based approach stems from increasing evidence that geography is an independent marker for these infections. For example, a person living in one of six syphilis clusters was over 20 times as likely as someone living outside a cluster area to have syphilis [13]. Furthermore, geographic syphilis clusters tend to cover larger areas and persist over time when compared to outbreak areas [14]. High-prevalence spatial clusters of HIV-infected persons have also been described, and residing in such clusters has been independently associated with elevated risk of acquiring HIV infection [15]–[16]. More recently, higher average community HIV viral load (CVL) has also been associated with higher neighborhood incident HIV infection [17]. TB “hot spots” have also been identified, with geographic clustering of cases attributed to overcrowding and tendency of immigrants to live in the same residential area [18], [19].

Recognizing geographic clustering of TB and sexually transmitted diseases has implications not only for investigating underlying etiology, but also for real-time intervention. In one study, the yield of a GIS-based approach to TB screening was higher compared to traditional screening methods [20]. Similar success has been reported with GIS-based screening for HIV infection [21], [22]. However, the value of an integrated (TB, HIV, and syphilis) screening approach has not been prospectively tested.

A GIS-based approach offering combined HIV, syphilis and TB screening would take advantage of shared elements of a core area “hot spot”: neighborhood sociocultural factors, local partner selection, and co-occurring high-risk behaviors, such as illicit drug use [14]. We therefore conducted a study of GIS-based screening (“GIS-THIS”: GIS-based Screening for Tuberculosis, HIV, and Syphilis) to ascertain the feasibility and yield of such a strategy.

## Methods

### Ethics Statement

The study protocol was approved by the Duke University Institutional Review Board as well as the Wake County Human Rights-Consumer Protection Committee, and written informed consent was obtained from each participant.

### GIS Mapping

The residences of incident TB, HIV, and syphilis cases in Wake County, North Carolina between 1/1/05–12/31/07 were mapped using ArcMap 9.3 GIS software (ESRI, Redlands, CA, USA). The geocoded addresses were transformed into a density map, using the Spatial Analyst Kernel Density Tool Areas. After data review, areas with the highest densities of all three diseases (defined as areas with greater than ten cases per square mile over the three-year period) were designated as “hot spots.” A map of Wake County streets was overlaid on the study map, to determine which street intersections and local businesses were located within the “hot spots”.

### Community Screenings

In collaboration with community nurses and disease intervention specialists from the HIV, syphilis, and tuberculosis clinics at the county health department, we identified community sites for local screenings, which were conducted 2/6/2009–3/11/2011. Sites were chosen based on location within a “hot spot,” availability of an area within the site to administer confidential surveys, and acceptability by the site owner. Community-based advertising and small incentives (snacks, beverage and a $5 grocery gift card) were used to attract participants. Persons were excluded from the study if they were younger than 18 years old or did not speak English or Spanish. Eligible participants were asked to complete a verbally administered questionnaire encompassing demographics, comorbidities, perceived risk of infection, and health care utilization, results of which have been described previously [23].

HIV testing was performed with ELISA and Western blot, and pooled specimens were sent for RNA quantification to detect acute HIV infection. Syphilis testing was performed with syphilis-specific IgG (Trepsure®) followed by the toluidine red unheated serum test (TRUST) for positive IgG results. Quantiferon Gold In-Tube® (QFT-GIT) (Cellestis Limited, Carnegie, Victoria, Australia) was used for TB testing; blood was placed in an incubator within 12 hours of phlebotomy, incubated for 16–24 hours, and processed according to manufacturer instructions by the Duke Clinical Immunology Laboratory. All three tests were performed with one blood draw.

### Result Notification and Follow-up

Participants were instructed to come to the health department or return to the screening site for test results. In cases of positive HIV or syphilis tests, participants were also contacted directly by a disease intervention specialist, who referred them to the health department for further management. Persons with a positive QFT-GIT were referred to the TB Clinic for a chest radiograph; those without evidence of active TB disease were offered free treatment for latent TB infection (LTBI) with four months of rifampin, a CDC-recommended regimen that has been associated with better tolerability and adherence than nine months of isoniazid [24]. Participants with positive tests were followed for six months to determine whether appropriate treatment was obtained.

### Clinic Screening

For a comparison group, HIV/syphilis and latent TB rates for the clinic populations at the Wake County STD and TB clinics were abstracted from documented laboratory results for patients presenting to the health department during the study time frame (2/2009–3/2011). The Wake County Health Department is physically located outside the identified disease “hot spots” described above. HIV and syphilis testing is offered to all patients who present to the STD clinic, and the most common reasons for presentation to the clinic include genitourinary symptoms, sexual contact with a partner recently diagnosed with an STD, and referral of high-risk persons by primary care providers or local employers based on demographic and medical risk factors. Latent TB testing is offered to all persons referred to the TB clinic, and the most common reasons for presentation to the clinic include close contact to a recently diagnosed active TB case, refugee screening, and referral by primary care providers or employers. All persons who undergo TB testing are routinely offered HIV testing (but not syphilis), and TB testing is not routinely offered to patients presenting to the STD clinic. Methods for testing HIV and syphilis were the same as those used for the community screenings (ELISA with confirmatory Western blot for HIV and Trepsure followed by TRUST for syphilis); for latent tuberculosis, persons who met Centers for Disease Control (CDC) criteria for a positive tuberculin skin test (TST) were defined as cases.

### Endpoints and Analysis

The primary outcome of the study was case detection rates of LTBI, syphilis, and HIV. Chi-square testing was used to compare rates from GIS-based community screening to those from Wake County TB and STD clinics during the same time period. Secondary outcomes included rates of treatment completion for persons with LTBI and syphilis, and entry into care (defined as a clinic visit with an HIV provider by six month follow-up) for persons with HIV.

## Results

### Identification of Hot Spots

**Figure 1:**
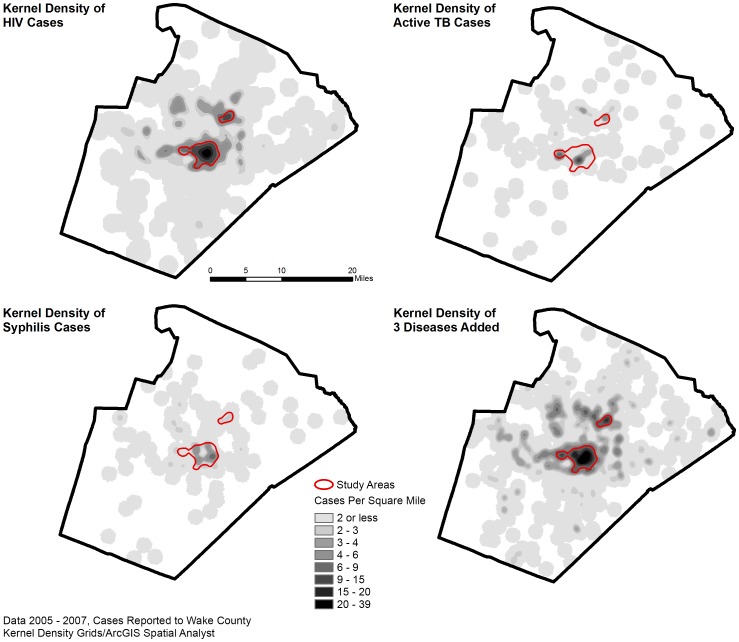
HIV, Syphilis and Active Tuberculosis Cases, 2005–2007. Based on public health surveillance data from January 2005 through December 2007, disease-specific cases were geocoded, including 665 HIV cases, 150 active TB cases, and 155 syphilis cases, with a match rate of 93%. An overlay map with data for all three diseases was used to identify two high-density “hot spots” (outlined).

**Figure 2:**
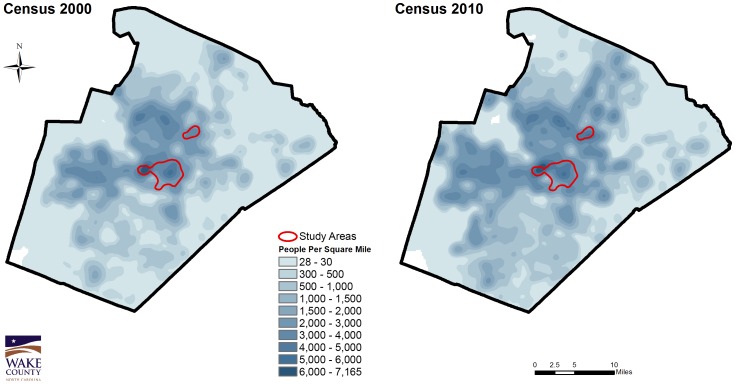
Population Density Maps, Wake County, NC by Census Blocks, 2000 and 2010. Kernel density maps generated from U.S. Census block centroids (2000 and 2010 data) demonstrate that population density alone (total population per square mile) does not reliably predict the identified disease “hot spots”.

A total of 150 active TB, 155 syphilis, and 665 HIV cases residing in Wake County, North Carolina were geocoded from 1/1/2005–12/31/2007. An overlay of maps for all three diseases identified two areas with highest density of all three diseases and were designated “hot spots” (Figure 1). This map was visually compared to U.S. census population density maps during the same time frame; high population density alone (total population per square mile) did not appear to correlate with the identified disease “hot spots” (Figure 2). From 2/2009 through 3/2011, 255 participant-encounters occurred through 16 community screenings conducted within the hot spots at ten different sites, including a church, grocery store, apartment complex, night club, and three community centers. Two participants were subsequently excluded from the study based on inadequate consent documentation, and one participant was excluded because of age. Three participants presented to more than one screening event; only their first encounter was included for analysis. A total of 247 participants were included in the analysis.

### Study Population

Ninety-five percent (n = 235) of study participant residential addresses were geocoded and demonstrated close proximity to the pre-specified “hot spots”(data not shown). For the other 12 participants, including homeless persons, two of the reported residences were P.O. boxes, and the remainder did not provide addresses. The majority of participants were African American men with a median age of 44 (Table 1). Participants reported high-risk behaviors: 66% were previously incarcerated, 39% had lived in a homeless shelter, 29% had a history of crack cocaine use, and 60% did not have a regular physician. However, 34% reported never having been tested for HIV, and 41% did not recall prior tuberculin skin testing. Based on survey responses, 67% participants perceived their risk of HIV or syphilis to be low, and 76% participants reported their risk of tuberculosis to be low. Sociodemographic information for the general population in the targeted hot spots, Wake County, and North Carolina state were also collected from the U.S. Census and are summarized in Table 2.

**Table 1:** Demographics and Behaviors of Study Participants.

Variable	Participants (N = 247), N (%)
Median age (IQR)	44 (20,32)
Race	
Black, Non-Hispanic	186 (75.3)
White, Non-Hispanic	12 (4.9)
Hispanic	42 (17.0)
Native American	3 (1.2)
Not reported	4 (1.6)
Male	143 (57.9)
Foreign-born	43 (17.4)
Diabetes	26 (10.5)
Hepatitis B or C (self-reported)	12 (4.9)
Prior incarceration	163 (66.0)
Homeless (ever)	96 (38.9)
Tobacco use (current or prior)	188 (76.1)
Alcohol abuse (≥3 drinks on average daily or ≥5 drinks on any day)	58 (23.5)
Drug use (current or prior)	
Crack	72 (29.1)
IV drugs	7 (2.8)
Prior STD	98 (39.7)
Unprotected sex in past year	150 (60.7)
Median (IQR) number of sexual partners in previous year (n = 135)	1 (1,3)
Median (IQR) number of sexual partners in lifetime (n = 127)	8 (3,20)

**Table 2:** Sociodemographic Characteristics of General Population in Study Areas, Wake County, and North Carolina.

Census Variable	Central Spot*	Northern Spot*	Wake County¥	North Carolina£
Total population	45,180	11,576	900,993	9,535,483
% Male	52.1%	48.3%	48.7%	48.7
Median Age (years)	28	28.7	34.4	37.4
% African American	48.2%	47.5%	20.7%	21.5%
% Hispanic or Latino	9.9%	31.2%	9.8%	8.4%
Average household size	2.21	2.56	2.55	2.48
% Vacant housing units	13.1%	7.7%	7%	13.5%
% Renters	68.7%	74.6%	34.9%	33.3%

* Source for Density Areas: ESRI Business Analyst, Census 2010 Summary Profile.

¥ Source for Wake County: http://www.wakegov.com/NR/rdonlyres/A51B919D-A7BC-48AC-92AC-2EF6FCEE60DD/0/2010CensusWakeCountyGeneralProfile.pdf.

£ Source for North Carolina: http://factfinder2.census.gov/faces/tableservices/jsf/pages/productview.xhtml?pid=DEC_10_DP_DPDP1&prodType=table.

### Feasibility

Ninety-seven percent (240/247) of participants had blood drawn (two persons left prior to blood draw and five persons had phlebotomy failures). All persons who had blood drawn had valid HIV results. One participant had an indeterminate syphilis IgG result. Three participants declined TB testing after consenting to the study, and three participants had laboratory or logistical errors, including insufficient blood volume in the Quantiferon tube (N = 2) or loss of sample during transport (N = 1).

### Case Detection Rates

HIV prevalence was significantly higher among study participants (3% [95% CI 1.4, 6.5]) than among persons screened at the Wake County STD Clinic from 2/2009–3/2011 (0.4% [95% CI 0.3, 0.5], p<0.001, personal communication Yvonne Torres, Table 3). Fifteen percent [95% CI 11.0, 21.7] of study participants had LTBI versus 6% [95% CI 5.6, 6.6] prevalence at the Wake County TB clinic 2/2009–3/2011 (p<0.001). 19% [95% CI 14.1, 24.4] had serologic evidence of previous or current syphilis infection, compared to 6% [95% CI 5.0, 6.0] in the Wake County STD clinic (7/2009–6/2010). Two patients were diagnosed with multiple infections: one with concurrent HIV and LTBI (see below), and one with HIV and syphilis. Furthermore, based on self-report and review of medical records, three of the eight HIV-infected participants were co-infected with hepatitis C, and a fourth was co-infected with hepatitis B.

**Table 3:** Measured Prevalence of HIV, Latent Tuberculosis, and Syphilis in Study Group Compared to Health Department Clinic Population.

Infection	Prevalence of Infection by Site of Screening % (95% CI)		P value
	*Community GIS Sites*	*Health Department Clinic*	
*HIV*	3.0 (1.4, 6.5)	0.4 (0.3, 0.5)	p<0.001
*Latent TB*	15.0 (11.0, 21.7)	6.0 (5.6, 6.6)	p<0.001
*Syphilis*	19.0 (14.1, 24.4)	6.0 (5.0, 6.0)	P<0.001

### Engagement in Care

**Figure 3:**
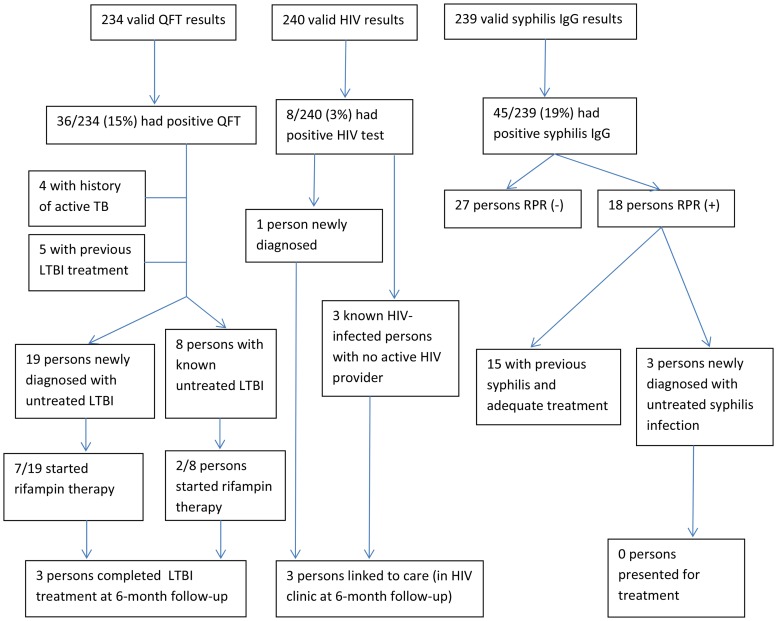
Yield and Follow-up from Geographic-based Screening for HIV, TB, and Syphilis. After six months of follow-up, three persons completed LTBI treatment, three persons with HIV were linked to care, and no patients presented for syphilis therapy

Of the eight participants identified with HIV, one was newly diagnosed. He entered into HIV care, was started on antiretrovirals, and remained in care at six-month follow-up. He was co-infected with LTBI, for which he completed therapy (rifampin). Three HIV-infected persons were previously diagnosed, but were not in care. Two of these reengaged and remained in care at six-month follow-up. Multiple attempts were made to contact the third without success. Four HIV-positive persons were already in care at the time of the screening; one died by six-month follow-up. Of the 36 patients identified with LTBI, 27 had not completed appropriate treatment. Fifteen (56%) presented for a chest radiograph to rule out active tuberculosis. Nine (33%) initiated therapy (Figure 3), and three of those completed treatment. Of the 45 patients with positive treponemal tests for syphilis, 18 (40%) also had a positive TRUST, and three of these patients did not recall or have any documented history of previous treatment. Two of these patients had a TRUST of 1∶2 and one had a TRUST of 1∶1, likely representing cases of latent syphilis. Repeated attempts to contact these patients were not successful by six-month follow-up. The 27 persons with discordant treponemal and nontreponemal test results were not offered therapy based on treatment history and absence of clinical symptoms.

### Stability of Hot Spots

To assess changes in case density over the course of the study, a case density map for incident TB cases between 2007–2009 was created and compared to the density map with cases from 2005–2007. There was no change in the location of the “hot spots” over the four-year study period (data not shown).

## Discussion

Integrated TB, HIV, and syphilis screening based on geospatial data has significant potential to increase the impact of screening programs organized by resource-limited health departments. The GIS-THIS study demonstrates that a geospatial approach to integrated screening is feasible and has potential to be at least complementary to clinic-based screening and perhaps higher-yield among underserved populations. An integrated screening approach using an interferon gamma release assay for the TB screening component allows a single blood draw to test for multiple infections in high risk individuals, permitting more efficient use of limited public health resources than separate disease-specific screening efforts.

Prevalence of HIV infection (3%) in our GIS-based screening cohort was higher than in the health department STD clinic and higher than overall U.S. prevalence (0.4%) [25]. This HIV prevalence is also notably higher than that observed in cohorts of emergency department patients and hospital inpatients, groups that have increasingly been targeted for routine HIV testing. In studies from Denver and Boston, HIV prevalence in emergency departments ranged from 0.3% to 0.6% [26], [27]. HIV screening has been deemed cost-effective in settings with >0.1% disease prevalence [28], but these analyses did not examine community-based outreach testing, which involves extra costs. A formal analysis of cost effectiveness of GIS-based integrated screening will be important to determine whether and under what conditions this tool should be integrated into public health practice.

Integrated testing was not only important for efficiency, but also to detect co-infection in our target population, which included individuals at high risk for all three infections. Integrated testing takes advantage of shared risk factors, and targets persons with multiple infections (e.g. HIV and latent tuberculosis) who may otherwise have poorer health outcomes than a person with either infection alone. Recognizing this fact, the Centers for Disease Control and Prevention launched the Program Collaboration and Service Integration (PCSI) initiative in 2009 with the explicit goal of fostering collaborative activities aimed at reducing HIV, viral hepatitis, STD, and TB infections [29]. The GIS-based integrated testing performed in this study demonstrated one method to promote the goals of the PCSI initiative by integrating multiple public health programs with geographic data.

The GIS-THIS study has a number of limitations. First, identified “hot spots” were assumed to remain in the same locations between the time the map was generated and when screening efforts occurred. The larger “hot spot” in the center of the county was, in fact, robust over time in terms of HIV and syphilis prevalence, based on a prior geographic analysis of the infections in 2000 [30], as well as our repeat mapping with data through 2009.

Second, due to financial limitations preventing use of QFT-GIT in the clinic and logistical constraints preventing use of TST in the field, TST was used for tuberculosis screening test in the clinic, while QFT-GIT was used at the community outreach sites. Given the lower specificity of TST as compared to QFT-GIT, however, this likely resulted in a higher number of positive tests at the clinic than would be expected were QFT-GIT used, suggesting that the increase in positive TB tests at the GIS sites would still be significant and is likely an underestimate.

This study may also have identified a higher number of newly diagnosed HIV infections if rapid HIV tests were used to attract participants. Such tests were not used due to inadequate staffing for posttest counseling and the potential for loss of confidentiality at community sites.

Third, a significant number of persons identified with HIV or LTBI in our population had been previously identified with these infections, so significant effort was expended to find previously known infections. However, many of these persons had either never engaged in appropriate care or had fallen out of care. An unanticipated benefit of the community-based screenings was reengagement of persons with known infections into care. While our study identified only one newly infected HIV patient, two persons with previously diagnosed HIV infection were reengaged in care as a result of these screenings. Similarly, of eight patients previously diagnosed with latent TB who previously refused or did not complete therapy, two accepted after this intervention, suggesting that multiple contacts to high-risk individuals may improve eradication efforts. The opportunity to reengage such persons in care using GIS-based screening efforts in the community is probably at least as important as finding new cases; linking individuals with positive tests to care has been shown to be more cost-effective than offering testing to additional people [31].

Despite the benefit of GIS-based screening in linking some individuals to appropriate healthcare, engagement in such healthcare was still suboptimal in our population. Of eight HIV-positive participants, five were under care of an HIV provider at six months of follow-up. This snapshot follow-up is incomplete, as patients may subsequently miss clinic visits or have problems with antiretroviral initiation or adherence. Utilization of medical resources early on, however, has been associated with improved virologic outcomes [32]. Similarly, of 27 patients with LTBI who were candidates for therapy, only 3 (11%) ultimately completed treatment. None of the patients diagnosed with untreated syphilis presented for therapy.

Of note, both individual demographic factors and geographic community factors could be implicated in our participant’s higher prevalence of infection as compared to the state as a whole. Persons in our study tended to be older and African American or Hispanic and came from areas with a higher proportion of renters (Table 2), a variable that has been associated with neighborhood instability [33].

In summary, the GIS-THIS study demonstrates the potential for geographic-based community outreach to provide integrated disease screening for high-risk populations, with reasonable yield and opportunities to reengage infected persons in healthcare. Continued innovation is needed to improve this approach; combining our geospatial approach with social networks may facilitate deeper penetration into the highest-risk populations. Furthermore, to make this a cost-effective and high-impact public health program, linkage to care must be improved. Interventions such as intensive case management programs and financial incentives show promise in engaging persons with LTBI and STDs in care [34], [35], [36], and should be studied in combination with GIS-based integrated screening in future studies.
